# Flood tolerance in two tree species that inhabit both the Amazonian floodplain and the dry Cerrado savanna of Brazil

**DOI:** 10.1093/aobpla/ply065

**Published:** 2018-10-12

**Authors:** Hérica Ribeiro Almeida Pires, Augusto Cesar Franco, Maria Teresa Fernandez Piedade, Veridiana Vizoni Scudeller, Bart Kruijt, Cristiane Silva Ferreira

**Affiliations:** 1 Department of Botany, University of Brasilia, Brasilia, DF, Brazil; 2 National Institute for Amazonian Research (INPA), INPA/Max-Planck Project, Manaus, AM, Brazil; 3 Federal University of Amazonas, Department of Biology, Manaus, AM, Brazil; 4 Wageningen Environmental Research (ALTERRA), Wageningen, The Netherlands

**Keywords:** Adaptation, environmental stress, flood tolerance, phenotypic plasticity, population differentiation, seed germination in water, submergence tolerance, waterlogging

## Abstract

Comparing plants of the same species thriving in flooded and non-flooded ecosystems helps to clarify the interplay between natural selection, phenotypic plasticity and stress adaptation. We focussed on responses of seeds and seedlings of *Genipa americana* and *Guazuma ulmifolia* to substrate waterlogging or total submergence. Both species are commonly found in floodplain forests of Central Amazonia and in seasonally dry savannas of Central Brazil (Cerrado). Although seeds of Amazonian and Cerrado *G. americana* were similar in size, the germination percentage of Cerrado seeds was decreased by submergence (3 cm water) and increased in Amazonian seeds. The seeds of Amazonian *G. ulmifolia* were heavier than Cerrado seeds, but germination of both types was unaffected by submergence. Three-month-old Amazonian and Cerrado seedlings of both species survived 30 days of waterlogging or submersion despite suffering significant inhibition in biomass especially if submerged. Shoot elongation was also arrested. Submersion triggered chlorosis and leaf abscission in Amazonian and Cerrado *G. ulmifolia* while waterlogging did so only in Cerrado seedlings. During 30 days of re-exposure to non-flooded conditions, *G. ulmifolia* plants that lost their leaves produced a replacement flush. However, they attained only half the plant dry mass of non-flooded plants. Both submerged and waterlogged *G. americana* retained their leaves. Consequently, plant dry mass after 30 days recovery was less depressed by these stresses than in *G. ulmifolia*. Small amounts of cortical aerenchyma were found in roots 2 cm from the tip of well-drained plants. The amount was increased by flooding. Waterlogging but not submergence promoted hypertrophy of lenticels at the stem base of both species and adventitious rooting in *G. ulmifolia*. Despite some loss of performance in dryland plants, flood tolerance traits were present in wetland and dryland populations of both species. They are part of an overall stress-response potential that permits flexible acclimation to locally flooded conditions.

## Teaser

Comparing plants of the same species thriving in flooded and non-flooded ecosystems helps to clarify the interplay between natural selection, plant plasticity and stress adaptation. We compared responses of seeds and seedlings of Genipa americana and Guazuma ulmifolia to substrate waterlogging or total submergence. Both species are commonly found in floodplain forests of Central Amazonia and in seasonally dry savannas of Central Brazil. We provide evidence that flood tolerance traits are also present in dryland populations, being part of an overall stress response potential that permits flexible acclimation to locally flooded conditions.

## Introduction

Trees of white-water river floodplain forests in Central Amazonia are subjected to extended periods of flooding and large variations in water level which can fluctuate 10 m or more (Junk *et al.* 1989). To survive these extreme flooding conditions, trees must be highly adapted to this habitat. Several studies have provided strong evidence that most of the genera and species making-up these seasonally flooded forests originated in non-flooded neighbouring forests, or in more distant dry or seasonally dry ecosystems (Kubitzki 1989; Worbes *et al.* 1992; Wittmann *et al.* 2013). The existence of populations of the same species occurring in flooded and non-flooded ecosystems provides an excellent opportunity for comparative studies of the mechanisms involved in flood tolerance and adaptation in plants and more broadly on impact of interactions between natural selection and phenotypic plasticity on survival and competitive success. Earlier studies have shown that tropical tree seedlings from neighbouring flooded and non-flooded populations of the same species do not respond in the same way to flooding (Ferreira *et al.* 2009, 2010; Silva *et al.* 2010). Here we focus on traits related to flood tolerance in *Genipa americana* L. (Rubiaceae) and *Guazuma ulmifolia* Lam. (Malvaceae), two tree species that are widely distributed throughout the neotropical region and are known to tolerate long periods of flooding (Santiago and Paoli 2007; Cortezi and Colli 2011). Both species are commonly found in Central Amazonian floodplain forests of Northern Brazil, but also occur in the seasonally dry upland savannas of Central Brazil. Because flooded forest and seasonally dry savanna populations of the two species are geographically segregrated, we expected large phenotypic differences between the two populations in their responses to long-term flooding.

Persistence and expansion of plant populations and recovery from disturbance are dependent on successful seed germination and seedling establishment. In seasonally flooded tropical forests, seeds of some tree species germinate and form seedlings as they float or even when totally underwater (Ferreira *et al.* 2007; Oliveira-Wittmann *et al.* 2010; De Melo *et al.* 2015). In contrast, seeds of other tree species are known to float for a shorter or longer period before sinking to the bottom, where they remain dormant until the flood water recedes (Kubitzki and Ziburski 1994; Oliveira-Wittmann *et al.* 2010; Lucas *et al.* 2012). Dormancy prior to the re-introduction of some oxygen as floodwater recedes may be advantageous for ensuring seed germination at this more favourable time. Seed germination in water reduces the time required for seedlings to establish and resume growth when waters finally recede (Ferreira *et al.* 2007, 2017; De Melo *et al.* 2015). However, they need to have sufficient energy reserves to sustain basal metabolism until the flood waters recede, thereby allowing normal photosynthesis and aerobic respiration to proceed.

Because of the broad amplitude of flooding to which Central Amazonian floodplain forests are exposed, newly established seedlings should be able to tolerate prolonged periods of waterlogging or submersion including the potentially harmful effects of low- and no-oxygen conditions (Pigliucci and Kolodynska 2002; Benz *et al.* 2007). The formation of adventitious roots, aerenchyma and lenticels in waterlogged plants may facilitate this tolerance by enabling transport of oxygen to the roots and rhizosphere (Kozlowski 1997; Waldhoff *et al.* 1998; Visser *et al.* 2003; Colmer and Voesenek 2009). Submerged plants are subjected to a much more serious risk of O_2_ deprivation. Although anatomical features that improve internal gas exchange would still be important, conservation of energy by regulation of metabolic processes becomes essential to ensure plant survival. Cessation of energy and carbon-demanding processes such as growth, leaf abscission, downregulation of non-essential processes to further conserve energy and induction of anaerobic energy production are typical metabolic plant responses to long-term submergence (Colmer and Voesenek 2009; Ferreira *et al.* 2009; Colmer *et al.* 2014). Additionally, traits that improve underwater photosynthesis under very low light conditions can significantly enhance both internal oxygen concentrations and carbohydrate contents, alleviating the risk of oxygen and carbon deprivation in completely submerged plants (Mommer and Visser 2005; Pedersen *et al.* 2013).

Flood tolerance would probably not play a major role in savanna populations of *G. americana* and *G. ulmifolia* since savannas of Central Brazil are mostly associated with deep well-drained nutrient-poor soils (Eiten 1972). The climate is seasonal, with a dry season that extends from May to September. By comparing the seed and seedling responses to flooding from these two origins, we aim to gain knowledge on phenotypic plasticity and adaptive evolution of traits related to flood tolerance and pose the following questions: Do floodplain and savanna populations of the two species respond in the same way to flooding in the early stages of the plant life cycle? More specifically, are changes in morphology and anatomy that would enhance species tolerance of flooding expressed solely among seedlings of floodplain populations? Are there trade‐offs in allocation between root and above‐ground biomass in response to long-term flooding?

In an effort to answer these questions, we examined seed germination in water and the effects of long-term flooding (30 days) on survival, growth, biomass accumulation and biomass distribution in the seedlings of *G. americana* and *G. ulmifolia* from Amazonian low-lying floodplain forests and from the seasonally dry savannas of Central Brazil. We also assessed seedling recovery upon removal from flooding and changes in morphology induced by flooding.

## Methods

### Species, fruit collection and seed processing

*Genipa americana* L. (Rubiaceae) and *G. ulmifolia* Lam. (Malvaceae) are widely distributed in Central and South America (Kubitzki 1989; Wittmann *et al.* 2013). The two species were sampled in two contrasting seasonal ecosystems that are about 1500 km apart: the flooded forests of Brazilian Central Amazonia and the seasonally dry savannas of Central Brazil. Fruits were collected from three to four trees each from different populations growing in sites characterized by native vegetation. Fruits from Central Amazonia were collected from low-lying floodplain forests in the islands of Xiborena (3°11′36.73″S; 59°56′33.34″W) and Marchantaria (3°1′28″S; 60°8′48”W) at the margins of the Solimões/Amazon River, near the city of Manaus. The height of the flood pulse (amplitude) in the sampled areas was about 5 m. Fruits from Central Brazil were collected in well-drained areas covered with savanna vegetation (cerrado *sensu lato*, Eiten 1972), in the municipality of Padre Bernardo, Goiás (15°25′03.37″S; 48°10′27.11″W) and in the Federal District (15°52′40.15″S; 47°57′07.77″W). For the purposes of this study, fruit collection sites will be referred to as Amazonia (flooded forests) or Cerrado (seasonaly dry savannas of Central Brazil or cerrado *sensu lato*).

Fruits were collected between June and August 2013, which coincided with the flooding period in the Amazonia and the dry period in the Cerrado. After collection (Registration for collection SISBIO 10257-1, Ministry of Environment, Water Resources and Legal Amazonia, Brazil), fruits were stored and transported in thermal boxes to the Laboratory of Plant Stress Physiology of the University of Brasilia (UnB) in the city of Brasília, Brazil, where seeds were extracted from the fruits and mixed together to obtain one single lot per ecosystem for each species. Seed size was measured as dry mass. Fifty seeds of each species and each ecosystem were lyophilized and then weighed individually on a 0.0001 g precision scale. For the germination trials in Experiment 1 and to obtain seedlings for Experiment 2, fresh seeds were previously disinfected with 2 % sodium hypochlorite for 15 min, which was then removed under running water. To break the dormancy of seeds of *G. ulmifolia* used in Experiment 2, seeds were immersed in boiling water for 1 min before planting to ensure uniform emergence of seedlings. Boiling water was not necessary for seeds of *G. american*a since they are non-dormant (De Souza *et al.* 1999; Queiroz *et al.* 2012).

### Experiment 1: seed germination and seedling development in water

To assess the influence of flooding on seed germination, a completely randomized design with two treatments (either flooded under 3 cm water or non-flooded), two origins and four replicates of 25 seeds each was used. Seeds were placed in transparent acrylic boxes with closed lids (11 cm × 11 cm × 3 cm) and kept in a B.O.D-type germination chamber (model MA402/1, Marconi^®^, Brazil) at 28 °C, with a 12-h photoperiod and photosynthetic photon flux density of 35 µmol m^−2^ s^−1^ delivered by white fluorescent lamps. Seeds were left to germinate for 60 days under the following two treatments: (i) non-flooded, with substrate consisting of moist double thickness filter paper and (ii) flooded, seeds submerged in 3-cm-deep distilled water. Dissolved oxygen concentration in the water was measured daily with a digital oximeter (Lutron, DO – 5519, Taipei, Taiwan) and averaged 3.2 ± 0.5 (Mean ± SD) mg L^−1^ at 28 ^o^C for the duration of the experiment. This is about one-third of the concentration in water in equilibrium with the air. Germination was scored as the emergence and curvature of the radicle (about 1.5 cm). Non-flooded, germinated seeds were individually transferred to plastic cups (200 mL) arranged in trays (43 cm × 28 cm × 5 cm) with damp but not saturated 1:1 mixture of Bioplant^®^ substrate (a commercial mix of coconut fibre and powder, pine bark, rice shell, vermiculite; NPK and micronutrients; pH 6.0–6.5) and vermiculite. Flooded, germinated seeds were transferred to plastic pots with distilled water (300 mL). They were kept under the same conditions of temperature and light as in the germination experiments and followed for 30 days to evaluate seedling emergence underwater defined as emergence of the caulicle and opening of the cotyledonary leaves. Formed seedlings were removed from water, planted in 1:1 mixture of Bioplant^®^ and vermiculite and monitored until new leaves were produced, when we considered seedling establishment to have occurred.

### Experiment 2: long-term flooding and recovery

Seeds of both species were placed to germinate in trays (43 cm × 28 cm × 5 cm) with vermiculite as substrate, and kept in a growth room under the same conditions of temperature and photoperiod of Experiment 1 but at a photosynthetic photon flux density of 20 µmol m^−2^ s^−1^. After emergence, seedlings were transferred to 500 mL plastic pots (one seedling per pot) containing Bioplant^®^ substrate and grown in a greenhouse (about 10 % of ambient light) until they reached the age of 90 days. Then they were subjected to the experimental flooding conditions.

For simulation of flooding events, a completely randomized design with three treatments, two origins and 20 replications was used (*N* = 120 plants per species). Each replication consisted of a single 50 L polyethylene container. Two individually potted plants, one of *G. americana* and one of *G. ulmifolia*, were placed inside each container. Plants were acclimated in these conditions for 1 week before the start of the experiment. The three treatments were: non-flooded (well-watered; substrate kept damp but not saturated), waterlogged (roots and lower part of the stem flooded, water height of about 2 cm above the level of the substrate) and submerged (whole plant submerged). Tap water was used and 15 mL of 5 % commercial sodium hypochlorite commercial solution was diluted in each litre of water applied to the flooded plants to prevent proliferation of mosquito larvae.

The experiment was conducted in the greenhouse and lasted for 30 days. Maximum and minimum air temperatures averaged 31 ^o^C and 17 ^o^C, respectively, whereas the photoperiod was around 13 h during the 30-day duration of the experiment. Dissolved oxygen concentration in the water was measured weekly at around 9 am in the waterlogged and submergence treatments. Values (mean ± SD) averaged 5.04 ± 0.25 mg L^−1^ at 23.4 ± 0.9 ^o^C and 5.11 ± 0.17 mg L^−1^ at 22.6 ± 0.6 ^o^C, in waterlogged and submerged conditions, respectively, and did not differ between treatments. Seedlings were inspected weekly for the appearance of lenticels and of adventitious roots on the lower stem. Shoot length (from the base to the tip of the stem) was measured and the number of leaves counted.

Fifteen plants per treatment of each species and origin were chosen at random and harvested at the end of the 30-day flooding period. A total of 120 plants was used for biomass determination (10 plants per treatment of each species and origin) while the other 60 plants were used for anatomical analysis (five plants per treatment of each species and origin). The remaining 60 potted plants were removed from the containers, allowing rapid free drainage and kept in the greenhouse, with daily watering to field capacity. Plant recovery and survival were monitored for a period of 30 days, after which plants were harvested for biomass determination. Shoot length was also recorded and the number of leaves counted. Plants for biomass determination were separated into roots, stem and leaves and parts stored in liquid nitrogen. Every sample was subsequently lyophilized for 48 h and weighed to 0.0001 g to determine dry mass.

For anatomical studies, secondary roots from the main root were collected, fixed in formalin–acetic acid–alcohol 70 % (FAA 70), and preserved in 70 % alcohol. Transverse sections of the secondary roots (approximately 2.0 cm from the root apex) were made with a hand-held microtome, dehydrated in a graded ethanol series, and stained with safranin:alcian blue 4:1 (Luque *et al.* 1996). The sections were mounted in synthetic resin (Paiva *et al.* 2006). Digital photographs were obtained with a DM 750 photomicroscope (Leica Microsystems Ltd, Switzerland) and analysed with the Leica LAZ EZ software version 2.0.0.

### Statistical analysis

For each species, we applied Model I two-way factorial analysis of variance with equal replication followed by Tukey’s test to evaluate the effects of seed origin and flooding on germination success and the effects of plant origin and flood regime on dry mass of leaves, stems and roots. A mixed model three-way ANOVA was used to test for the effects of plant origin (Amazonia and Cerrado), flood regime and sampling date and interactions on shoot elongation and number of leaves. Plant origin and flood regime were considered as fixed factors. A Box–Cox transformation was applied to achieve normality and homogeneity of variances for number of leaves and shoot elongation of *G. ulmifolia* and a square-root transformation for shoot elongation of *G. americana*. For all tests, differences were considered to be significant at *P* < 0.05.

## Results

### Seed size and germination

Seeds of *G. americana* from the Amazonia and the Cerrado did not differ in dry mass, averaging 53.0 ± 0.01 mg (mean ± SD) and 52.0 ± 0.01 mg, respectively. In contrast, Amazonian seeds of *G. ulmifolia* had significantly higher mass (6.0 ± 0.00 mg) than those from the Cerrado (5.0 ± 0.00 mg).

**Figure 1.**
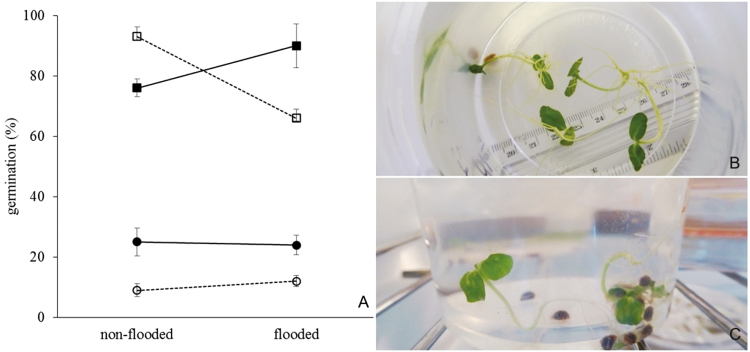
Effect of flooding on the percentage germination (number of germinating seeds in relation to the total number of seeds) of *Genipa americana* and *Guazuma ulmifolia* from Amazonian floodplain forests or from seasonally dry savannas of Central Brazil (Cerrado) (A). Data expressed as mean ± standard error. The interaction origin × flood level was significant (*P* = 0.001) for *G. americana*, while only the origin (*P* = 0.002) had a significant effect for *G. ulmifolia*. *Genipa americana*: (filled square) Amazonia, (open square) Cerrado; *G. ulmifolia:* (filled circle) Amazonia, (open circle) Cerrado. Seedlings of Amazonian *G. americana* (B) and of Cerrado *G. ulmifolia* (C) developed in water.

ANOVA results revealed that the interaction flooding × origin was significant for seed germination of *G. americana* (Fig. 1A). Seeds of *G. americana* showed high germination percentage (>75 %) under non-flooded conditions with higher values for Cerrado seeds (Fig. 1A). Flooding increased the germination percentage of Amazonian seeds by 17 % but decreased germination of Cerrado seeds by almost 30 %. Percentage germination was much lower for *G. ulmifolia*. Less than 30 % of the seeds of a given origin or treatment germinated and flooding was without effect on germination percentage. However, there was a significant effect of seed origin (Fig. 1A). Seeds of *G. ulmifolia* of Amazonian origin had double the germination percentage of those from the Cerrado.

All seeds that germinated in either flooded and non-flooded conditions developed into seedlings within a 30-day period following germination when grown in illuminated conditions (Fig. 1B and C). All underwater-formed seedlings survived when removed from water and planted in unsaturated substrate.

### Flooding effects on survival, growth, biomass accumulation and distribution

**Figure 2.**
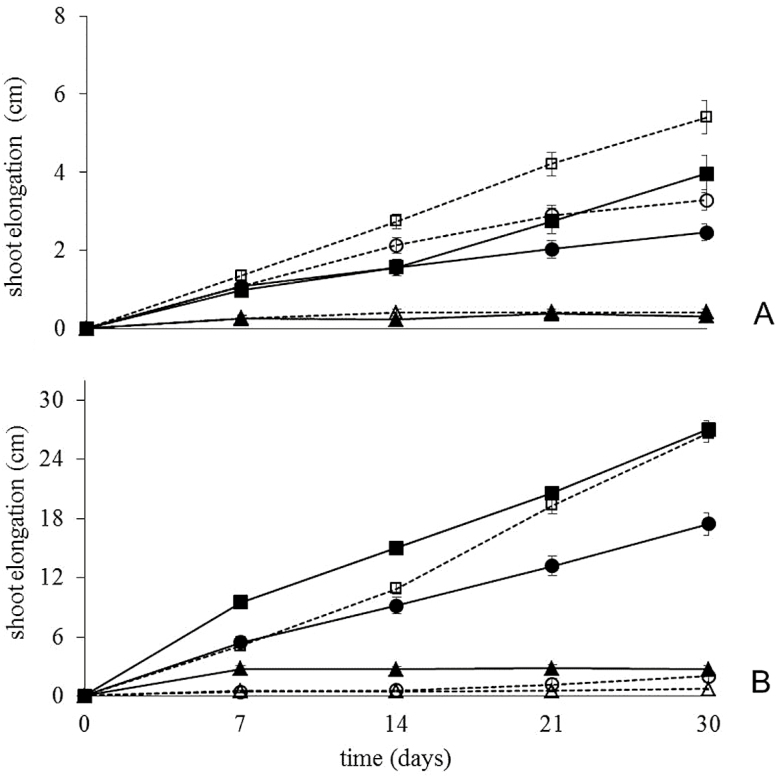
Shoot elongation of Amazonian (AM) and Cerrado (CE) plants of *Genipa americana* (A) and *Guazuma ulmifolia* (B) subjected to 30 days of flooding. Data expressed as mean ± standard error, *n* = 20 plants. (filled squares) Non-flooded-AM; (filled circles) waterlogged-AM; (filled triangles) submerged-AM; (open squares) non-flooded-CE; (open circles) waterlogged-CE, (open triangles) submerged-CE.

Non-flooded plants of both species continuously increased in shoot length. *Genipa americana* from the Cerrado showed faster rates of shoot elongation than those from Amazonia under well-drained conditions, while shoots of Amazonian and Cerrado derived *G. ulmifolia* elongated similarly over the 30 days of the experiment (Fig. 2). All plants of *G. americana* and *G. ulmifolia* survived the 30-day period of waterlogging or submersion. Nonetheless, both treatments had negative effects on growth of both species (Fig. 2). The flood level × origin and flood level × time interactions were significant for *G. americana*, while flood level × origin, flood level × time and origin × time interactions were significant for *G. ulmifolia* (Table 1). Irrespective of origin, seedlings of *G. americana* were able to elongate while waterlogged, but about 39 % slower than non-flooded plants. In *G. ulmifolia*, waterlogging was much more inhibitory in Cerrado seedlings (stem elongation reduced by 90 % after 30 days) than in Amazonian seedlings (shoot elongation reduced by only 35 %). When seedlings were subjected to complete submersion, shoot elongation was arrested in seedlings of both species regardless of origin (Fig. 2), although Amazonian *G. ulmifolia* showed a small increment in shoot length in the first week underwater.

**Table 1.** Results of the analysis of variance on the influence of population origin (Amazonia or Cerrado) and flood level on the number of leaves and shoot elongation of *Genipa americana* and *Guazuma ulmifolia*. ^a^Square-root transformed values for *G. americana* and Box-Cox for *G. ulmifolia*. ^b^Box-Cox transformed values for *G. ulmifolia.*

Model term	*G. americana*			*G. ulmifolia*		
	d.f	SS	*P*	d.f	SS	*P*
Shoot elongation^a^						
Origin	1	0.2	<0.001	1	14.95	<0.001
Flood level	2	5.1	<0.001	2	33.93	<0.001
Time	1	1.2	<0.001	1	4.31	<0.001
Origin × flood level	2	0.5	0.046	2	6.07	<0.001
Origin × time	1	0.0	0.129	1	0.13	0.033
Flood level × time	2	0.6	<0.001	2	1.66	<0.001
Origin × flood level × time	2	0.0	0.780	2	0.06	0.334
Error	12	0.1		12	0.28	
Number of leaves^b^						
Origin	1	2.3	<0.001	1	549.7	<0.001
Flood level	2	10.7	<0.001	2	396.5	<0.001
Time	1	2.4	<0.001	1	18.1	0.002
Origin ×flood level	2	0.7	0.001	2	80.6	<0.001
Origin × time	1	0.4	0.003	1	6.8	0.039
Flood level × time	2	0.9	<0.001	2	214.8	<0.001
Origin × flood level × time	2	0.1	0.264	2	36.1	<0.001
Error	18	0.6		18	24.7	

**Figure 3.**
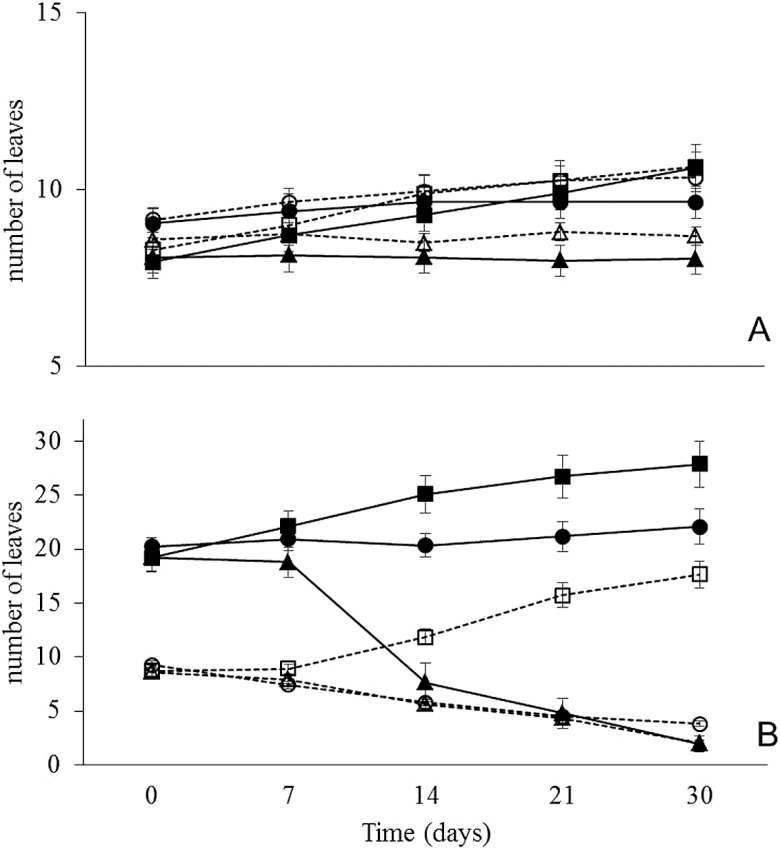
Total number of leaves present in Amazonian (AM) and Cerrado (CE) plants of *Genipa americana* (A) and *Guazuma ulmifolia* (B) subjected to 30 days of flooding. Data expressed as mean ± standard error, *n* = 20 plants. (Filled squares) non-flooded-AM; (filled circles) waterlogged-AM; (filled triangles) submerged-AM; (open squares) non-flooded-CE; (open circles) waterlogged-CE; (open triangles) submerged-CE.

At the beginning of the experiment, Amazonian and Cerrado *G. americana* had a similar number of leaves (around 8), while Amazonian *G. ulmifolia* had about two times the number of leaves of Cerrado plants (Fig. 3). The two species differed in patterns of leaf dynamics during the 30-day experiment (Fig. 3). The flood level × origin, flood level × time and origin × time interactions were significant for *G. americana*, while the flood level × time × origin interaction was significant for *G. ulmifolia* (Table 1). Non-flooded *G. americana* added 1–2 leaves per plant (Fig. 3A). In contrast, non-flooded *G. ulmifolia* added approximately 8–9 leaves per plant (Fig. 3B). In *G. americana* and regardless of the origin of the plants, neither waterlogging nor submergence promoted leaf loss (Fig. 3A) and there were no signs of leaf chlorosis even after 30 days of stress. This was not the case for *G. ulmifolia.* Although waterlogging did not result in leaf injury or leaf loss for Amazonian plants of *G. ulmifolia*, waterlogged Cerrado plants developed leaf chlorosis and had lost >75 % of the leaves after 30 days. When totally submerged both Amazonian and Cerrado plants of *G. ulmifolia* developed leaf chlorosis and had lost about 75 % (Cerrado plants) and >90 % (Amazonian plants) of their leaves by the end of the experiment (Fig. 3B).

In *G. americana* from both Amazonia and the Cerrado, total biomass was reduced by both flooding treatments (Tables 2 and 3), but especially strongly by submergence (−48 % in Amazonian plants, −55 % in Cerrado plants). In *G. ulmifolia*, total biomass was also decreased by flooding (Tables 2 and 3) especially by submergence (−80 % in Amazonian plants, −60 % in Cerrado plants). Cerrado plants were more adversally affected by waterlogging than were Amazonian plants (−55 % vs. −33 %). Overall, *G. ulmifolia* was more inhibited than *G. americana* by the flooding stresses, and this arose primarily through a greater susceptibility of stem and leaves relative to the roots, as shown by the significant increase in root:shoot ratios of submerged plants. In leaves, 75–93 % less dry mass was found in submerged plants of *G. ulmifolia* compared with well-drained ones. Flooding did not influence root:shoot ratios of *G. americana*, despite clear differences in root:shoot ratios between Amazonian and Cerrado plants of this species.

**Table 2.** Results of the analysis of variance on the influence of population origin (Amazonia or Cerrado) and flood level on biomass distribution of *Genipa americana* and *Guazuma ulmifolia*. Plants were harvested 30 days after the start of the watering regime.

Model term	*G. americana*			*G. ulmifolia*		
	d.f	SS	*P*	d.f	SS	*P*
Total biomass						
Origin	1	0.3	0.401	1	15.6	<0.001
Flood level	2	22.1	<0.001	2	175.5	<0.001
Origin × flood level	2	0.6	0.544	2	20.9	<0.001
Error	54	26.1		54	29.4	
Root mass						
Origin	1	0.3	<0.001	1	0.3	0.016
Flood level	2	0.3	<0.001	2	5.5	<0.001
Origin × flood level	2	0.1	0.090	2	1.1	<0.001
Error	54	0.7		54	2.9	
Stem mass						
Origin	1	0.5	0.001	1	3.7	<0.001
Flood level	2	1.1	<0.001	2	19.7	<0.001
Origin × flood level	2	0.3	0.057	2	2.2	0.001
Error	54	2.5		54	7.5	
Leaf mass						
Origin	1	0.4	0.130	1	2.1	<0.001
Flood level	2	10.9	<0.001	2	42.5	<0.001
Origin × flood level	2	0.0	0.921	2	4.5	<0.001
Error	54	9.9		54	6.0	
Root: shoot ratio						
Origin	1	0.6	<0.001	1	0.2	0.463
Flood level	2	0.0	0.024	2	1.9	<0.001
Origin × flood level	2	0.0	0.434	2	0.1	0.392
Error	54	0.1		54	1.8	

**Table 3.** Biomass distribution in Amazonian and Cerrado plants of *Genipa americana* and *Guazuma ulmifolia* in response to waterlogging and complete submersion. Each cell is the mean ± standard error of 10 plants harvested 30 days after the start of the watering regime. Different uppercase letters within the same column indicate significant differences between Amazonian and Cerrado plants, while different lowercase letters within the same row indicate significant differences across the three hydric conditions.

	*G. americana*			*G. ulmifolia*		
	Well-watered	Waterlogged	Submerged	Well-watered	Waterlogged	Submerged
Total biomass (g)						
Amazonia	2.78 ± 0.48 Aa	2.06 ± 0.50 Ab	1.45 ± 0.25 Ac	6.67 ± 1.00 Aa	4.44 ± 0.80 Ab	1.36 ± 0.21 Ac
Cerrado	2.97 ± 0.94 Aa	2.32 ± 0.85 Aa	1.34 ± 0.37 Ab	5.03 ± 0.44 Ba	2.38 ± 0.76 Bb	2.00 ± 0.69 Bb
Root mass (g)						
Amazonia	0.26 ± 0.07 Aa	0.18 ± 0.06 Ab	0.17 ± 0.06 Ab	1.56 ± 0.30 Aa	0.97 ± 0.14 Ab	0.54 ± 0.11 Ac
Cerrado	0.47 ± 0.14 Ba	0.32 ± 0.17 Bab	0.22 ± 0.10 Ab	1.17 ± 0.10 Ba	0.69 ± 0.27 Bb	0.77 ± 0.21 Bb
Stem mass (g)						
Amazonia	0.45 ± 0.15 Aa	0.42 ± 0.13 Aa	0.28 ± 0.07 Ab	2.41 ± 0.45 Aa	1.70 ± 0.41 Ab	0.65 ± 0.15 Ac
Cerrado	0.77 ± 0.34 Ba	0.66 ± 0.29 Ba	0.29 ± 0.08 Ab	1.72 ± 0.41 Ba	0.86 ± 0.36 Bb	0.68 ± 0.25 Ab
Leaf mass (g)						
Amazonia	2.06 ± 0.36 Aa	1.45 ± 0.34 Ab	0.99 ± 0.19 Ac	2.70 ± 0.36 Aa	1.77 ± 0.38 Ab	0.18 ± 0.24 Ac
Cerrado	1.84 ± 0.57 Aa	1.34 ± 0.58 Aab	0.82 ± 0.24 Ab	2.14 ± 0.20 Ba	0.84 ± 0.28 Bb	0.54 ± 0.39 Bb
Root:shoot ratio						
Amazonia	0.11 ± 0.03 Aab	0.10 ± 0.03 Aa	0.14 ± 0.04 Ab	0.31 ± 0.04 Aa	0.29 ± 0.05 Aa	0.72 ± 0.26 Ab
Cerrado	0.19 ± 0.04 Ba	0.16 ± 0.05 Ba	0.19 ± 0.06 Ba	0.30 ± 0.03 Aa	0.42 ± 0.13 Ba	0.70 ± 0.31 Ab

### Anatomical and morphological responses

**Figure 4.**
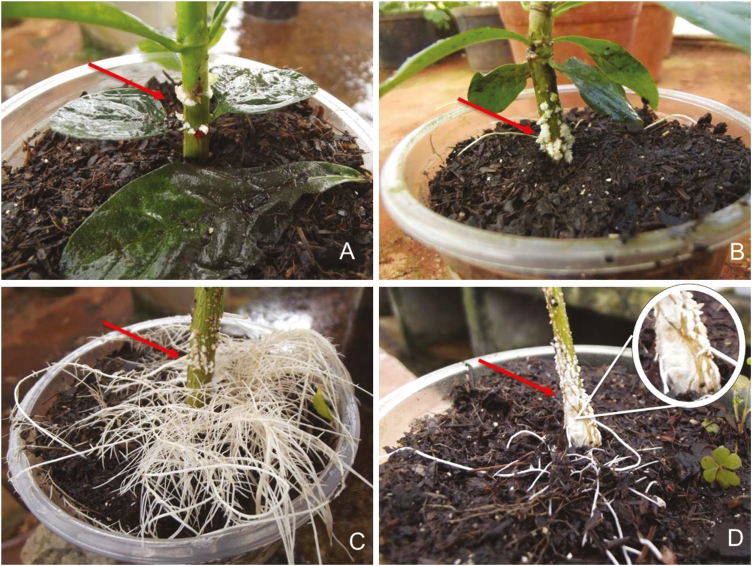
Hypertrophic lenticels (arrows) on stems of Amazonian (A) and Cerrado (B) *Genipa americana* and Amazonian (C) and Cerrado (D) *Guazuma ulmifolia* after being subjected to 30 days of waterlogging. Note also the presence of adventitious roots on stems of *Guazuma* (C and D). Insert in (D) shows the cortical cracks on the surface of the stem of Cerrado *Guazuma*.

Waterlogging induced the formation of hypertrophied lenticels at the stem base in both Amazonian and Cerrado seedlings of both species (Fig. 4A–D). They were observed after 7 days of waterlogging in *G. ulmifolia* and after 21 days of waterlogging in *G. americana*. Most (65 %) waterlogged Cerrado plants of *G. ulmifolia* developed hypertrophic, whitish spongy stems with cortical cracks, which were not present in Amazonian plants of this species (Fig. 4C and D) or in *G. americana* (Fig. 4A and B). Adventitious roots were formed in stems of waterlogged *G. ulmifolia* from the Cerrado (35 % of the plants) and Amazonia (100 % of the plants), but not in *G. americana* (Fig. 4A–D). Adventitious roots, hypertrophic stems or lenticels were not formed in non-flooded or submerged plants of either species.

**Figure 5.**
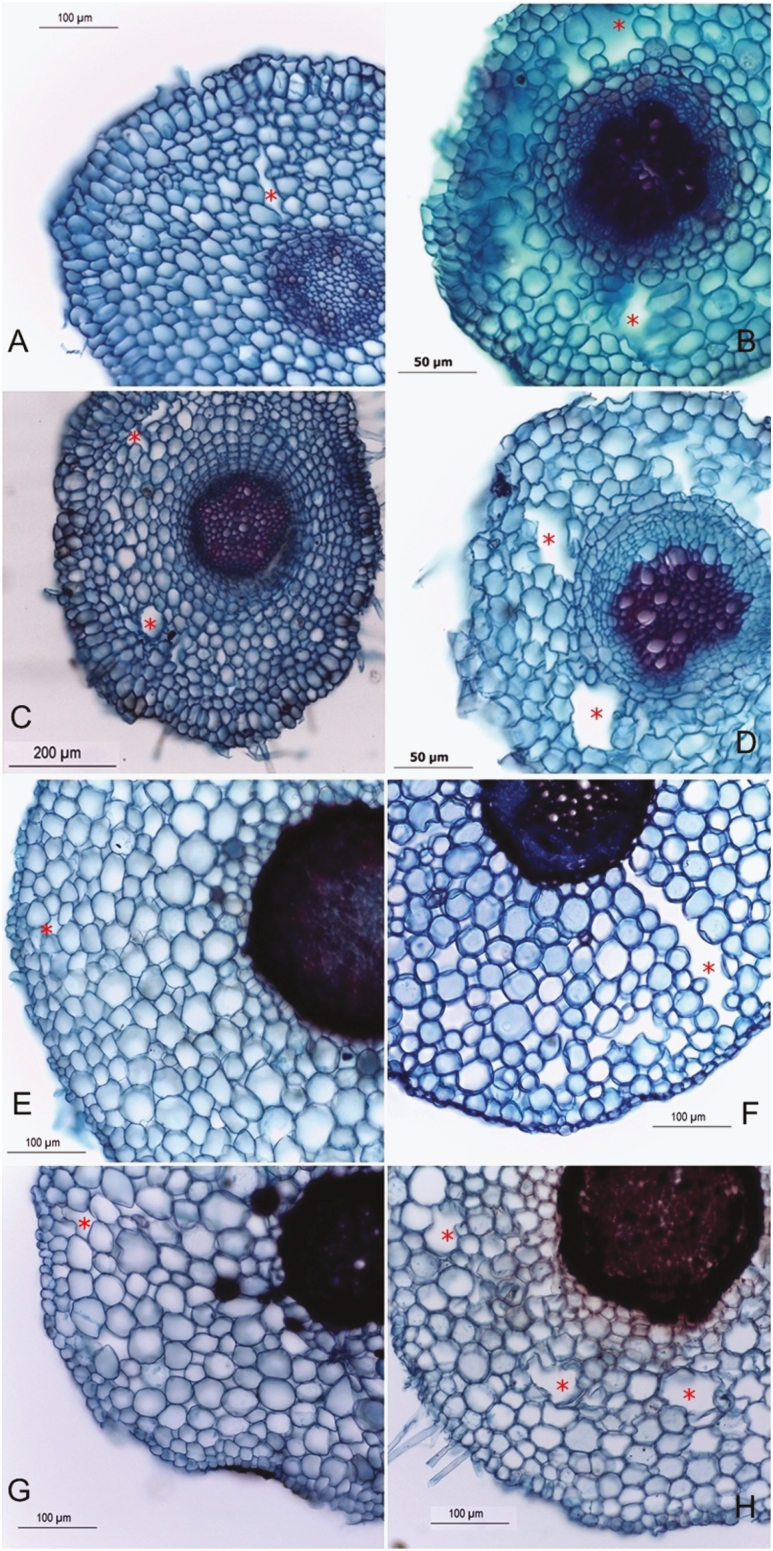
Transverse section of secondary roots of *Genipa americana* (A–D) and *Guazuma ulmifolia* (E–H). The red stars indicate enlarging air spaces in the root cortex of Amazonian (A,B,E,F) and Cerrado (C,D,G,H) plants kept well-watered (A,C,E,G) and after 30 days of complete submersion (B, D, F, H).

The anatomical analysis revealed the presence of small amounts of intercellular air spaces in roots of non-flooded seedlings of *G. americana* and *G. ulmifolia* from the Amazonia and the Cerrado (Fig. 5A, C, E, G). The air spaces were more prominent in *G. americana*. This tissue was more developed in submerged seedlings particularly in *G. americana* (Fig. 5B, D, F and H).

### Recovery after flooding

Regardless of origin and flooding level, all plants were alive 30 days after substrate drainage. Amazonian and Cerrado plants of *G. americana* recovered similarly (Table 4). They resumed growth and increased plant biomass (Tables 3 and 5). Overall, there was a residual treatment effect on total plant biomass after 30 days of re-exposure to non-flooded conditions (Table 4). Waterlogged Amazonian and Cerrado plants had 20 and 26 %, respectively, less total dry mass than non-flooded plants 1 month after they were removed from flooding and kept in well-watered conditions (Table 5). Submerged plants were more affected (−31 % total dry mass in Amazonian plants, −40 % in Cerrado plants). These differences were small and not discerned by post hoc Tukey’s pair-wise comparisons because of limitations in the sample size. Well-watered and waterlogged Cerrado plants of *G. americana* continued to invest relatively more in roots than the Amazonian plants as shown by their higher root:shoot ratios, but not the submerged plants (Tables 4 and 5).

**Table 4.** Results of the analysis of variance on the influence of population origin (Amazonia or Cerrado) and flood level on shoot length, number of leaves and biomass distribution of *Genipa americana* and *Guazuma ulmifolia*. Plants were kept for 30 days in well-watered conditions after being subjected to 30 days of flooding (waterlogging or complete submersion). ^a^Box-Cox transformed values.

Model term	*G. americana*			*G. ulmifolia*		
	d.f	SS	*P*	d.f	SS	*P*
Shoot length (cm)						
Origin	1	25.21	0.176	1	199.7	0.054
Flood level	2	138.52	0.012	2	5187.5	<0.001
Origin × flood level	2	6.23	0.786	2	506.2	0.013
Error	24	311.7		24	1168.0	
Number of leaves^a^						
Origin	1	0.000	0.482	1	23.27	<0.001
Flood level	2	0.000	0.779	2	8.94	0.003
Origin × flood level	2	0.000	0.977	2	1.66	0.263
Error	24	0.002		24	14.15	
Total biomass						
Origin	1	0.040	0.615	1	1.231	<0.001
Flood level	2	1.391	0.021	2	3.118	<0.001
Origin × flood level	2	0.097	0.731	2	0.045	0.048
Error	24	3.678		24	1.577	
Root mass						
Origin	1	0.280	0.104	1	0.470	0.038
Flood level	2	1.348	0.004	2	3.221	<0.001
Origin × flood level	2	0.373	0.171	2	0.638	0.056
Error	24	2.352		24	2.350	
Stem mass						
Origin	1	0.013	0.796	1	2.492	<0.001
Flood level	2	2.263	0.007	2	5.744	<0.001
Origin × flood level	2	0.015	0.962	2	0.874	0.014
Error	24	4.492		24	2.031	
Leaf mass						
Origin	1	0.037	0.609	1	1.198	0.001
Flood level	2	0.641	0.121	2	1.495	0.001
Origin × flood level	2	0.077	0.761	2	0.235	0.228
Error	24	3.324		24	1.788	
Root: shoot ratio						
Origin	1	0.158	0.016	1	0.541	<0.001
Flood level	2	0.031	0.523	2	0.009	0.860
Origin × flood level	2	0.151	0.057	2	0.060	0.373
Error	24	0.561		24	0.697	

**Table 5.** Shoot length, number of leaves and biomass distribution in Amazonian and Cerrado plants of *Genipa americana* and *Guazuma ulmifolia* that were kept in well-watered conditions after being subjected to 30 days of flooding (waterlogging or complete submersion). Each cell is the mean ± standard error of five harvested plants taken 30 days after the start of the well-watered conditions. Different uppercase letters within the same column indicate significant differences between Amazonian and Cerrado plants, while different lowercase letters within the same row indicate significant differences across the three hydric conditions.

	*G. americana*			*G. ulmifolia*		
	Well-watered	Waterlogged	Submerged	Well-watered	Waterlogged	Submerged
Shoot length (cm)						
Amazonia	13.7 ± 5.5 Aa	11.5 ± 2.5 Aa	8.1 ± 1.2 Aa	69 ± 4.4 Aa	60.2 ± 5.6 Aa	34.6 ± 7.8 Ab
Cerrado	15.8 ± 3.1 Aa	12.1 ± 1.4 Aa	10.9 ± 3.6 Aa	67.32 ± 8.8 Aa	43.7 ± 6.0 Bb	37.3 ± 2.8 Ab
Number of leaves						
Amazonia	10.6 ± 1.5 Aa	11.0 ± 3.6 Aa	10 ± 1.3 Aa	32.8 ± 10.5 Aa	22.6 ± 2.8 Aa	40.4 ± 15.8 Aa
Cerrado	11.4 ± 2.2 Aa	11.4 ± 2.8 Aa	10.4 ± 1.5 Aa	18.8 ± 2.6 Ba	7.2 ± 1.6 Bb	14.8 ± 8.1 Bab
Total biomass (g)						
Amazonia	5.28 ± 1.6 Aa	4.23 ± 0.6 Aa	3.66 ± 0.8 Aa	10.86 ± 1.2 Aa	7.78 ± 1.2 Ab	5.14 ± 0.9 Ac
Cerrado	6.22 ± 2.0 Aa	4.63 ± 0.7 Aa	3.73 ± 1.7 Aa	8.84 ± 1.5 Aa	3.74 ± 1.4 Bb	4.15 ± 1.8 Ab
Root mass (g)						
Amazonia	0.97 ± 0.2 Aa	0.85 ± 0.2 Aa	0.75 ± 0.1 Aa	3.30 ± 0.3 Aa	2.55 ± 0.7 Aa	1.70 ± 0.3 Ab
Cerrado	1.57 ± 0.6 Ba	1.03 ± 0.1 Aab	0.76 ± 0.3 Ab	3.47 ± 0.7 Aa	1.38 ± 0.5 Bb	1.63 ± 0.9 Ab
Stem mass (g)						
Amazonia	1.67 ± 0.8 Aa	1.15 ± 0.1 Aab	0.80 ± 0.2 Ab	4.92 ± 0.8 Aa	3.30 ± 0.6 Aa	1.48 ± 0.4 Ab
Cerrado	1.51 ± 0.6 Aa	1.19 ± 0.2 Aa	0.87 ± 0.5 Aa	3.13 ± 1.0 Ba	1.20 ± 0.4 Bb	1.18 ± 0.3 Ab
Leaf mass (g)						
Amazonia	2.64 ± 0.8 Aa	2.23 ± 0.4 Aa	2.11 ± 0.5 Aa	2.63 ± 0.4 Aa	1.94 ± 0.2 Ab	1.97 ± 0.2 Ab
Cerrado	3.14 ± 1.0 Aa	2.42 ± 0.4 Aa	2.11 ± 0.9 Aa	2.24 ± 0.3 Aa	1.16 ± 0.5 Bb	1.34 ± 0.6 Aab
Root:shoot ratio						
Amazonia	0.24 ± 0.04 Aa	0.25 ± 0.02 Aa	0.27 ± 0.05 Aa	0.44 ± 0.07 Aa	0.48 ± 0.08 Aa	0.49 ± 0.02Aa
Cerrado	0.35 ± 0.07 Ba	0.29 ± 0.02 Bab	0.26 ± 0.05 Ab	0.65 ± 0.09 Ba	0.58 ± 0.09 Aa	0.62 ± 0.12 Aa

Flooded plants of *G. ulmifolia* did not fully recover plant biomass after 30 days of re-exposure to non-flooding conditions, even though they had resumed stem growth and produced a new flush of leaves. The effects of flooding on biomass acummulation were largest for submerged Amazonian and Cerrado plants and waterlogged Cerrado plants (Tables 4 and 5). Those still averaged about half of the biomass of non-flooded plants after 30 days of re-exposure to non-flooding conditions. Waterlogged Amazonian plants of *G. ulmifolia*, which kept their leaves during exposure to waterlogging, were less affected. As with well-watered and waterlogged *G. americana*, root:shoot ratios were higher for Cerrado plants.

## Discussion

The two species showed contrasting responses to long-term flooding and recovery that were dependent on the degree of flooding and plant origin.

Amazonian and Cerrado *G. americana* had inherently higher germination percentages than *G. ulmifolia* in well-aerated moist substrate. Seeds of *G. americana* are known to be non-dormant, photoblastic neutral (De Souza *et al.* 1999) with high percentage germination and fast germination rates (Andrade *et al.* 2000; Queiroz *et al.* 2012; Salla *et al.* 2016) ensuring rapid seedling establishment if the seed is dispersed to favourable microsites. *G. americana* seeds of both origins also germinated and developed successfully into seedlings under water. Cerrado seeds showed lower inherent germination success in water than in moist substrate. Germination of Amazonian seeds was slightly enhanced under water, indicating a localized adaptation to annual flooding. It is already known that, after germination in water, seedlings of *G. americana* endure submersion for up to 90 days and still survive. This ability favours fast early growth of water-dispersed seedlings during the period of receding water in Amazonian floodplains (De Melo *et al.* 2015). Mature fruits and seeds of *G. americana* tend to sink and seed germination is prevented beneath a water column of 8 cm (De Souza *et al.* 1999). However, per cent seed germination was shown to be high (>80 %) after seeds were kept in water for 4 months, although germination significantly decreased thereafter. Induced dormancy in deeply submerged seeds might allow the formation of an ephemeral, submerged seed bank, ensuring seed survival and rapid seedling development during the period of receding water (Kozlowski 1984; Scarano and Crawford 1992; Kubitzki and Ziburski 1994).

*Guazuma ulmifolia* has more orthodox seeds that possess physical dormancy (Araújo Neto and Aguiar 2000; Sobrinho *et al.* 2012). This explains the low germination of Amazonian and Cerrado seeds of this species in the present experiments. Seeds of Amazonian origin had double the germination percentage of those of Cerrado origin, suggesting a lower physical resistance of the Amazonian seed coat. Drought-prone environments tend to favour species with dormant seeds (Allen and Meyer 1998; Jurado and Flores 2005). However, dormancy is not only a mechanism to prevent germination during unsuitable ecological conditions but it does allow more time for dispersal. By distributing the offspring through time, it minimizes the risk of population mortality in environments that are both harsh and unpredictable (Willis *et al.* 2014). Amazonian *G. ulmifolia* seeds also had a larger mass than Cerrado seeds, which was consistent with the larger size attained by their seedlings. Amazonian and Cerrado *G. americana* did not differ in seed and seedling size. Seed size is generally positively related to seedling size and seedling survival (Jakobsson and Eriksson 2000; Baraloto *et al.* 2005). It is suprising that unlike in *G. americana*, germination by seed from the Cerrado was not inhibited by flooding, suggesting that the species may have spread to drier regions from flood-prone Amazonia and retained this adaptive wetland feature.

The adverse effects of flooding on biomass accumulation were larger in seedlings of *G. ulmifolia* especially in submerged plants compared with *G. americana*. This is partially explained by the two species having distinct leaf phenology responses to flooding. Submerged *G. ulmifolia* thus behaved as a facultatively deciduous species because the stress triggered leaf chlorosis and premature leaf abscission thus inducing much leaf loss. This also applies to waterlogged Cerrado plants of *G. ulmifolia* . This leaf loss probably depressed the recovery of plant biomass following re-exposure to non-flooded conditions, because a new flush of leaves had to be produced first. This would have further consumed available carbon reserves. Leaf shedding in response to drought or flooding is a common strategy adopted of many woody species. It plays an important role in maintaining the hydraulic integrity of the stem and also reducing energy consumption (Kozlowski 1997; Parolin 2009; Pivovaroff *et al.* 2014). In general, it is preceded by nutrient reabsorption, which results in leaf chlorosis and senescence followed by shedding. When environmental conditions become favourable, new leaves are formed and growth resumes (Schöngart *et al.* 2002; Parolin *et al.* 2010). Although new flush of leaves was produced by Amazonian *G. ulmifolia* upon initial release from waterlogging, plants still averaged only 60 % of the biomass of non-flooded plants after 30 days recovery. In contrast, waterlogged *G. ulmifolia* of Amazonian origin retained much more foliage than waterlogged Cerrado plants (lost only 32 % of its leaves compared with 62 %) and this conferred a much stronger recovery in terms of biomass in the 30 days following drainage (28.4 % compared with 57.7 % in Cerrado). Retention of presumably functional leaves during waterlogging is seen as an adaptation to inundation that appears to have been lost by plants occupying dry Cerrado conditions.

The behaviour of *G. americana* was typical of evergreen species, maintaining its foliage even under submersion, which allowed rapid post-stress recovery.

Overall and despite the adverse effects of waterlogging or submergence on biomass accumulation, plants added much biomass following re-exposure to well-watered conditions. In waterlogged plants, the retention of leaves would have played a role in restoring the aerobic metabolism and in offsetting the costs of maintenance of metabolism under conditions of long-term root hypoxia. Leaf maintainance in submerged plants (as seen in both forms of *G. americana*) would allow a fast recovery of photosynthetic carbon assimilation and replenishment of the used carbon reserves as soon as the leaves are re-exposed to air (Pedersen *et al.* 2013). Depending on light availability, underwater photosynthesis can alleviate the adverse effects of submergence in many plants (Mommer and Visser 2005; Pedersen *et al.* 2013). Evergreen trees, such as *Symeria paniculata*, commonly found in floodplain areas of the Amazonia, can preserve the photosynthetic apparatus intact for long periods of time under water, without any signs of damage when the leaf lamina is re-exposed to aerated environments (Fernandez *et al.* 1999; Waldhoff *et al.* 2002; Parolin 2009). This especially seems to be the case in *G. americana* where submerged plants retained their leaves and recovered more strongly than submerged *G. ulmifolia* which lost most of its leaves during submergence. Trade-offs between deciduous and evergreen behaviour have been extensively studied in response to seasonal drought (for instance Wright *et al.* 2004; van Ommen Kloeke *et al.* 2012), or to waterlogging (Parolin 2001; Schöngart *et al.* 2002). More studies are needed to address better the trade-offs related to these two strategies in submerged conditions in which CO_2_ and O_2_ partial pressures are low and light is strongly limiting or unavailable and following draining of the soil. In the present study, leaf retention during waterlogging or submergence is linked to stronger recovery from these stresses.

In general, species with some degree of tolerance to waterlogging undergo anatomical modifications such as the development of aerenchyma in the secondary roots, and the formation of lenticels and adventitious roots on the stem. These structures are known to facilitate gas exchange between aerial and submerged plant parts, alleviating the hypoxic conditions and contributing to the recovery and maintenance of aerobic respiration in waterlogged seedlings (Kozlowski 1997; Shimamura *et al.* 2003; Jackson *et al.* 2009). Cerrado plants of *G. ulmifolia* developed cracks on the surface of the stems, causing rupture of the bark and exposing the internal tissues. This would have the same function of enhancing aeration by connecting the internal tissues directly with the atmosphere (Metcalfe 1931; Davanso *et al.* 2003; Shimamura *et al.* 2010; Oliveira *et al.* 2015). This response from the dryland-adapted form does not fit well with the notion of it being an adaptation to waterlogging.

Unlike waterlogging, submersion did not promote adventitious roots, hypertrophic stems or lenticels in either species. When all parts of a plant are underwater, gas exchange with the atmosphere is strongly curtailed and those morphological structures may be unable to develop as a consequence. Even if they did develop their function in totally submerged plants is difficult to envisage (Ferreira *et al.* 2009; Parolin 2009). Plant survival will depend on the availability and efficient use of internal carbon reserves that are associated with metabolic adjustments pertaining the co-ordinated downregulation of oxidative pathways and increasing anaerobic metabolism (Medina *et al.* 2009; Piedade *et al.* 2010). Inhibition of growth in non-aquatic plants under complete submergence, as shown by *G. americana* and *G. ulmifolia*, becomes essential to reduce the consumption of carbon and energy reserves (Ferreira *et al.* 2009; Parolin 2009).

The seeming presence of some constitutive aerenchyma in roots of non-flooded plants of *G. americana* and *G. ulmifolia* and the additional enlargement of the intercellular air spaces in flooded plants highlights the likely importance of this feature in enhancing plant tolerance to flooding. These observations need confirming by more extensive quantitative analyses. Plants subjected to drought conditions can respond with aerenchyma development (Zhu *et al.* 2010), because in addition to connecting and oxygenating tissues, it reduces tissue density, promoting a more efficient carbon use in plant metabolism (Drew *et al.* 2000; Zhu *et al.* 2010). Cerrado plants of both species also invested more in root biomass than the ones of Amazonian origin. High root:shoot ratios is a typical response to drought, low nutrient availability and fire, which characterize the savannas of Central Brazil (Moreira and Klink 2000; Hoffmann and Franco 2003).

## Conclusions

*Genipa americana* and *G. ulmifolia* are common trees in both the annually flooded forests of Amazonia and the drier non-flooded Cerrado of Central Brazil. When germinated underwater or when seedlings were grown for 30 days in waterlogged soil or under total submergence both species survived and grew on successfully following drainage. Differences in performance between species and between plants derived from seeds collected in Amazonia and the Cerrado indicated contrasting strategies for survival of flooding stress. However, flood tolerance traits were strongly expressed in both wetland and dryland populations of *G. americana* and *G. ulmifolia* despite some loss of performance in material from the Cerrado such as some inhibition of germination by flooding in *G. americana*, greater inhibition of shoot elongation and greater leaf loss in waterlogged *G. ulmifolia*. Our results provide evidence that flood tolerance traits are part of an overall stress response potential that permits flexible acclimation to locally flooded conditions. On the other hand, a potential role of these features in allowing *G. americana* and *G. ulmifolia* to successfully colonize the seasonally dry Cerrado of Central Brazil remains to be determined.

## Sources of Funding

This work was supported by Fundação de Apoio à Pesquisa do Distrito Federal (FAPDF, grant numbers 649/2015, 1078/2016), Conselho Nacional de Desenvolvimento Científico e Tecnológico (CNPq, grant numbers 308182/2015-4, 310547/2016-4), Programa Nacional de Apoio e Desenvolvimento da Botânica (PNADB-CAPES, AUXPE 451/2010), the C.T. de Wit Graduate School for Production Ecology & Resource Conservation, Wageningen University (082PE&RC2016), PELD MAUA/CNPq/FAPEAM (441590/2016-0). H.R.A.P. received a Master Scholarship from CAPES, Brazil.

## Contributions by the Authors

C.S.F., A.C.F. and M.T.F.P. conceived and designed the study. H.R.A.P. performed the experiments and collected the data (with assistance from C.S.F. and A.C.F.). C.S.F., A.C.F. and H.R.A.P. analysed and interpreted the data. C.S.F. and A.C.F. wrote the first draft. All authors discussed the results and contributed comments to the manuscript. All authors approved the final version of the manuscript.

## Conflict of Interest

None declared.
